# ROCK Inhibition as Potential Target for Treatment of Pulmonary Hypertension

**DOI:** 10.3390/cells10071648

**Published:** 2021-06-30

**Authors:** Tadeu L. Montagnoli, Jaqueline S. da Silva, Susumu Z. Sudo, Aimeé D. Santos, Gabriel F. Gomide, Mauro P. L. de Sá, Gisele Zapata-Sudo

**Affiliations:** 1 Programa de Pesquisa em Desenvolvimento de Fármacos, Instituto de Ciências Biomédicas, Universidade Federal do Rio de Janeiro, Rio de Janeiro 21941-902, Brazil; tmontagnoli@gmail.com (T.L.M.); ssjck@hotmail.com (J.S.d.S.); aimeediogeness@edu.unirio.br (A.D.S.); gabrielfonseca1508@gmail.com (G.F.G.); 2 Hospital Municipal Miguel Couto, Rio de Janeiro 22430-160, Brazil; susumu_sudo@hotmail.com (S.Z.S.); 3 Programa de Pós-graduação em Ciências Cirúrgicas, Faculdade de Medicina, Universidade Federal do Rio de Janeiro, Rio de Janeiro 21941-902, Brazil; 4 Instituto do Coração Edson Saad, Hospital Universitário, Faculdade de Medicina, Universidade Federal do Rio de Janeiro, Rio de Janeiro 21941-902, Brazil; mauropaesleme.cirurgia@gmail.com

**Keywords:** ROCK, pulmonary hypertension, right ventricle dysfunction

## Abstract

Pulmonary hypertension (PH) is a cardiovascular disease caused by extensive vascular remodeling in the lungs, which ultimately leads to death in consequence of right ventricle (RV) failure. While current drugs for PH therapy address the sustained vasoconstriction, no agent effectively targets vascular cell proliferation and tissue inflammation. Rho-associated protein kinases (ROCKs) emerged in the last few decades as promising targets for PH therapy, since ROCK inhibitors demonstrated significant anti-remodeling and anti-inflammatory effects. In this review, current aspects of ROCK inhibition therapy are discussed in relation to the treatment of PH and RV dysfunction, from cell biology to preclinical and clinical studies.

## 1. Pulmonary Hypertension: Clinical Features and Limitations of Approved Drugs

Pulmonary hypertension (PH) is a chronic disease of the cardiopulmonary system of multiple etiologies that presents a mean pulmonary arterial pressure (mPAP) above 20 mmHg [1]. Patients with PH have symptoms related to right ventricle (RV) dysfunction, resulting in precordial pain, exertion dyspnea, exercise intolerance, fatigue, syncope and edema [2]. Although echocardiographic examination may indicate the presence of PH, the direct measurement of mPAP by cardiac catheterization remains the gold standard for diagnosis [3]. PH is caused by exacerbated cell proliferation, fibrosis and tone dysregulation in pulmonary arterioles, phenomena that result in a progressive increase in pulmonary vascular resistance [4,5]. The pathogenesis of PH initiates in the pulmonary circulation but RV dysfunction is the major cause of increased morbidity and mortality. Therapeutic strategies focus drugs that target the molecules involved in the regulation of pulmonary vascular tone to reduce the burden in the RV, thus preventing the progression of heart failure and premature death [5]. The current treatment of PH aims to improve hemodynamic parameters and exercise tolerance, which result in increased patient survival [6]. After the confirmation of PH diagnosis using RV catheterization, the response to the vasodilator test and risk stratification are used in order to define the best treatment strategy. When the pulmonary vascular reactivity test is positive, calcium channel blockers (CCBs) is the option, but when the test is negative or the response to CCB is not efficient, the stratification guides the best specific therapeutic choice for PH.

The pathophysiological mechanisms of PH involve dynamic interactions between the different cells of the pulmonary vessels, which constitute the main determinant of cellular dysfunction of the pulmonary arteries due to changes in proliferation, apoptosis, differentiation, migration and survival. Vascular remodeling in the lungs of patients with PH occurs with the abnormal hyperproliferation of vascular smooth muscle cells (VSMCs) in response to altered signaling by growth factors [7,8]. The deregulation in cell cycle control in VSMCs and the excessive release of growth factors by endothelial cells are abnormalities intrinsically linked to the pathogenesis of PH [9]. In fact, the cells of the pulmonary arterial vasculature, including endothelial cells, develop an anti-apoptotic and pro-proliferative phenotype [10].

The inflammatory process is a factor that reinforces the pro-proliferative process and fibrosis of the pulmonary arteries [11]. Apoptosis can be a triggering mechanism for PH by causing the structural degeneration of endothelial cells that induces the appearance of apoptosis-resistant endothelial cells and hyperproliferative phenotype [12]. Inflammation plays an important role during the development of PH because some patients have increased serum levels of tumor necrosis factor alpha (TNF-α), interleukin (IL)-12 and -6, and interferon-γ, indicating the value of the reversal of pulmonary vessel injury through an anti-inflammatory effect [13]. The pharmacological treatment of PH is primarily based on five classes of drugs: prostacyclin (PGI_2_) receptor agonists, PGI_2_ analogues, endothelin (ET-1) receptor antagonists, soluble guanylyl cyclase (sGC) stimulators and phosphodiesterase (PDE)-5 inhibitors [14]. Those drugs act on the pulmonary vasculature by modulating the signaling pathways that interfere with increased vascular tone, promoting vasodilation and leading to a reduction in pulmonary resistance, which results in decreased RV overload [15].

Despite advances in the treatment of PH, the estimated survival of patients is approximately 5–7 years, even after the beginning of therapy with clinically available drugs [16]. Despite providing improvement in RV function, exercise capacity, quality of life and reduction of mortality, current treatments do not have a significant effect on vascular remodeling and inflammation [17] and although some of these drugs prolong the survival of patients with PH, they do not interrupt or reverse the disease progression [18]. Thus, the identification of new therapeutic agents is extremely important, which will interfere with signaling pathways involved in the exacerbated cell proliferation of pulmonary vessels and to promote cardioprotection, reducing the mortality of PH. Rho-associated protein kinases (ROCK) are relevant for PH pathogenesis and progression, because of their interference on pro- and anti-proliferative pathways [19].

## 2. Overview on ROCK Structure and Functions

ROCKs are the most well studied effectors of the small GTPase RhoA, which affect cellular functions mainly by modulating the arrangement of the actin cytoskeleton. These enzymes belong to the AGC family of serine/threonine protein kinases and exist in two isoforms: ROCK1 (also named ROKβ or p160^ROCK^), encoded by gene *ROCK1* on locus 18q11.1 and first identified in human platelet extracts [20] and ROCK2 (also named ROKα or Rho-kinase) encoded by the gene *ROCK2* on locus 2p25.1 and isolated from rat brain extracts [21]. In mammals, both ROCK isoforms are ubiquitous but ROCK1 predominates in the kidney, spleen, liver, and ROCK2 in the brain and the heart [22,23]. This distribution suggests different functions, as described below [24]. Both isoforms of ROCK possess a similar tridimensional structure composed of three main regions: an N-terminal kinase domain, a Coiled-coil region containing the Rho-binding domain (RBD) and a C-terminal Pleckstrin homology domain (PHD), with an internal cysteine-rich zinc-finger domain [25]. The homology between ROCK1 and ROCK2 is equivalent to 65% along the entire protein but reaches over 90% in the kinase domain and they are almost identical in the ATP-binding site [23,24,25]. The kinase domain of ROCK is generally thought to be active, as indicated after cleavage by caspase-3 (for ROCK1) or granzyme B (for ROCK2), despite the slight catalytic activity of the enzyme. This effect results from an interaction between both the N- and C-terminus, resulting in autoinhibition [23,25]. The binding of phosphatidic lipids or arachidonic acid to the PHD or interaction of GTP-bound RhoA with the RBD, anchor the enzyme in the plasma membrane and increase the phosphorylation of intracellular ROCK substrates [23,24,25].

As effectors of Rho GTPases, ROCKs regulate cytoskeletal responses to extracellular stimuli and modify cell contractility, motility, proliferation and morphology. ROCK modulates actin filament assembly resulting in force generation and cell adhesion, migration and phagocytosis. The activation of ROCK is also involved in the contraction of the actomyosin ring and in the intermediate filaments disorder during cytokinesis [26,27]. Besides the assembly of F-actin stress fibers, ROCK also mediates the release of transcription factors such as myocardin-related transcription factor (MRTF) and yes-associated protein (YAP), promoting changes in gene expression and phenotypic changes [26,27]. Control over gene transcription by the ROCK direct phosphorylation of interferon response factors (IRFs) is also reported [27]. Finally, ROCK can also promote survival, by stimulating autophagy, and cell proliferation, by mediating the G1/S transition [27].

## 3. Cellular Effects of ROCK on the Cardiovascular System

As effector of the GTPase RhoA, ROCKs modulate cell morphology and the formation of stress fibers and focal adhesions in different cellular models. The subsequent development of selective inhibitors and genomic approaches further evidenced that the regulation of actin cytoskeleton by ROCK not only influences cell biomechanics but also profoundly affects cell signaling. In addition, both pharmacological and molecular biology strategies also led to the identification of cell-specific effects mediated by ROCK isoforms involved in neuronal, endocrine and cardiovascular physiology and disease [23]. The main purpose of this section is the description of the impact of ROCK activation to cell biology, which contributes to PH and RV failure.

### 3.1. Vascular Smooth Muscle Cells (VSMC)

Pulmonary artery vasoconstriction and remodeling are factors responsible for the increased vascular resistance seen in patients with PH [28]. The abnormal balance in vascular smooth muscle cell (VSMC) hypertrophy, excessive proliferation and apoptosis results in the formation of the characteristic angio-proliferative lesions found in PH [29]. PH can induce the increased expression and activity of ROCK in the lung vasculature of patients and in rodent models of primary or secondary PH [29,30]. The activation of ROCK plays an important role in regulating the VSMC structure and function and mediating signaling pathways involved in their migration, proliferation and apoptosis. Sustained vasoconstriction in response to endogenous chemical (vasoconstrictors, hypoxia) or physical stimuli (stretching) can explain the increased vascular tone in pulmonary arteries. In VSMCs, the activation of ROCK by agonists such as angiotensin-II, endothelin-1 and thromboxane A2, leads to MLCP inhibition and enhances the contraction at the submaximal intracellular Ca^2+^ concentration (calcium sensitization) [31,32,33]. This mechanism also contributes to tone control in response to hypoxia (hypoxic pulmonary vasoconstriction) or increased intraluminal pressure (myogenic tone) [31,32]. In addition, the relation between ROCK and hypoxia inducible factor (HIF)-1α may further aggravate pulmonary vasoconstriction [34]. Therefore, the usefulness of ROCK inhibitors as pulmonary artery vasodilators was demonstrated by their activity using different vasoconstrictor stimuli [35,36,37]. VSMC-specific *ROCK2* knockdown mice displayed preserved RV systolic pressure after exposure to hypoxia, indicating an important role for ROCK2 in vasoconstriction induced by PH, as previously indicated by increased serum ROCK2 activity in PH patients [32,33,38,39].

Considering the intense VSMC contraction and proliferation, a role for oxidative stress is suggested in the pathogenesis of PH [30]. The production of reactive oxygen species in pulmonary arteries by NADPH oxidase (NOX) is reported to enhance vasoconstriction in response to chronic hypoxia, in part by activating the ROCK calcium sensitization of actomyosin filaments [30,40]. In rodent models, the implication of the NOX/ROCK pathway in VSMC proliferation was confirmed [33,41] and the production of reactive oxygen species was exacerbated by increased ROCK-induced cyclophilin A secretion [33].

The prolonged mechanical stress in VSMCs may trigger different adaptive mechanisms, which culminate in their differentiation towards a phenotype with increased migratory and proliferative capacities and resistance to apoptosis instead of the quiescent contractile phenotype [23,42,43]. Direct stimulation by known VSMC mitogens, which includes platelet-derived growth factor (PDGF), interleukin (IL)-6 or leptin, promotes VSMC dedifferentiation towards a hyperproliferative phenotype [44,45]. The activation of ROCK by PDGF results in the disruption of the antiproliferative signaling of bone morphogenetic protein (BMP)-2/Smad-1 mediated by the extracellular signal-regulated kinase (ERK) [30,34,46,47]. In addition, ROCK promotes the YAP-mediated inhibition of the BMP receptor (BMPR)-2/Inhibitor of DNA binding (ID)-1 pathway. ROCK also downregulates p27^Kip1^, an endogenous CDK inhibitor, further promoting cell proliferation [48,49,50]. In contrast, gene knockout or the inhibition of ROCK demonstrated the total reversal of the hyperproliferative phenotype of VSMCs [33,46,47]. The major contribution of isoform ROCK2 for proliferative lesions observed in animal models of PH was demonstrated in knockout mice [33,38,39]. VSMCs also show an increased migratory profile, which may contribute to neointimal lesions and the neomuscularization of distal arterioles. The modulation of cytoskeletal dynamics by ROCK1 is directly involved in cell migration [30,47].

### 3.2. Endothelial Cells

Endothelial cells (ECs) are involved in the maintenance of pulmonary vascular homeostasis by releasing paracrine factors and modulating the permeability of the endothelial barrier to leukocytes [23,51]. EC dysfunction is associated with progression of PH, especially in the generation of plexiform vascular lesions [33]. Moreover, cardiomyocyte hypoxic damage, apoptosis and cardiac inflammation and fibrosis occurs because of the dysfunction of ECs, since they are directly implicated in providing proper oxygen supply to cardiac cells [23,52]. Interestingly, PH patients also have RV capillary rarefaction, especially in the presence of underlying autoimmune disorders, such as systemic sclerosis [53].

ECs contribute to vascular tone regulation by producing vasoconstrictor (endothelin-1) or vasodilator factors (PGI_2_, nitric oxide). Although they influence the pulmonary artery, VSMCs are the main target for current therapy of PH, which does not benefit all patients. The endothelial dysfunction seen in PH involves lower nitric oxide (NO) production by the reduced expression and activation of endothelial NO synthase (eNOS) [23]. Gene knockdown of ROCK or its inhibition can upregulate eNOS expression in ECs by controlling the degradation of *NOS3* mRNA [23,32,52]. Moreover, the inhibition of ROCK also increases eNOS phosphorylation by Akt, enhancing its catalytic activity, presumably by reducing the activation of phosphatase and tensin homologue (PTEN) [23,48,54]. Additionally, ROCK may also impair NO bioavailability by promoting oxidative stress in ECs [55].

Under increased shear stress, activated ECs may behave as immune cells, performing the synthesis and secretion of inflammatory mediators [43]. Endothelial activation in PH involves the development of a pro-inflammatory phenotype with the increased expression of cell adhesion proteins and production of cytokines, via NFκB-stimulated transcription, thus enhancing immune cell recruitment and infiltration in the vessel wall [43,48,56]. Intimal fibrosis may also improve leukocyte-EC interaction in response to changes in stiffness during extracellular matrix remodeling [57]. ROCK activation plays an important role because the use of fasudil or Y-27632 abolished EC activation and leukocyte adhesion and migration [56,57]. Thrombin and inflammatory cytokines produced, respectively, by platelets and immune cells may increase ROCK activity in ECs, establishing a positive feedback, which exacerbates endothelial inflammation [32,43].

The activation of ROCK in ECs also mediates cytoskeleton contraction and the loss of intercellular junctions, resulting in the disruption of the endothelial barrier [33,58,59,60]. Although both ROCK isoforms seem to be involved in the endothelial expression of adhesion molecules and leukocyte migration, ROCK2 controls vascular permeability during immune cell diapedesis [61]. Moreover, the use of ROCK inhibitors demonstrated protective effects by reversing such alterations and preserving the barrier integrity both in vitro and in vivo [56,59,62,63]. Since these intracellular events are also involved in endothelial-to-mesenchymal transition (EndMT), ROCK promotes EC detachment and migration from the intimal layer. In fact, crucial signaling pathways for EndMT, such as those initiated by transforming growth factor (TGF)-β, SNAIL and SLUG, all converge to ROCK activation and its inhibition may significantly reduce vascular and cardiac remodeling [23,64].

Resistance to apoptosis and increased proliferation observed in response to vascular inflammation and hypoxia are remarkable features of ECs in PH [65]. Apoptotic insults mediated by ROCK may select apoptosis-resistant cells [59,66,67]. Proliferation in pulmonary artery EC cultures induced by hypoxia involves the increase in cyclin A and cyclin D1 in order to promote cell cycle progression. Under these conditions, the use of Y-27632 or a small-interfering RNA targeting ROCK2 abolished EC proliferation without compromising cell viability, indicating that ROCK inhibition may also control EC phenotypic changes seen in PH [68]. These effects could reflect ROCK activation by vascular growth factor and EC stiffening, as ROCK inhibitors also block EC migration and angiogenesis [69,70,71].

### 3.3. Cardiomyocytes

The importance of RV dysfunction reflects its role as a predictor of mortality in patients with PH caused by either heart or lung diseases [52,72]. The mechanisms involved in RV dysfunction and remodeling remain poorly understood and seem to include alterations in sarcomere structure and function, excitation–contraction coupling and cardiomyocyte metabolism, along with increased oxidative stress, apoptosis, fibrosis and inflammation [52].

Cardiac hypertrophy is an important adaptive response to increased afterload with the involvement of activated ROCK. The increase in sarcomere assembly and in the expression of fetal genes observed in cardiomyocytes exposed to different agonists, such as angiotensin-II, endothelin-1, and leptin, is abolished by transfection with dominant negative ROCK or direct ROCK inhibition by Y-27632 [39,48,73,74,75]. Animal models provided further insight on ROCK contribution to cardiac hypertrophy after ischemia or increased mechanical stress [48,76]. The conditional overexpression of a dominant-negative Rho-kinase in mouse hearts recovered RV dysfunction, hypertrophy and fibrosis and improved survival in response to pressure overload [24,76,77]. Wild type mice submitted to pulmonary artery constriction displayed increased content of the isoform ROCK2 in the RV, indicating its greater relevance to RV failure [24,39,76]. In fact, while both isoforms participate in cardiomyocyte apoptosis and fibrosis, the heart-specific knockout or global knockdown of ROCK2 demonstrated its relevance for cardiac hypertrophic responses [24,38,54,76,78,79]. The increased expression of ROCK2 in RV tissues also contributes to cardiac dysfunction and reduced ventricle-arterial coupling, as evidenced in animal models of PH and mechanical overload [76,77,80]. Since cardiac substrates of ROCK encompass multiple cytoskeleton-associated proteins, it may not be surprising that its activation modifies the contraction strength in cardiomyocytes [81]. In the heart, the inhibition of MLCP by ROCK underlies the calcium sensitization of myosin filaments, although its contribution to cardiac dysfunction seems irrelevant [81,82]. However, ROCK2 also phosphorylates cardiac troponin I (cTnI) and troponin T (cTnT) and inhibits the troponin complex, thus reducing the Ca^2+^-elicited development of tension [39,81]. The activation of ROCK under mechanical or metabolic stress promotes the function of sarcolemma proteins involved in cardiac action potential propagation and the generation of intracellular Ca^2+^ transients [83,84,85,86,87]. Therefore, ROCK inhibition may represent a strategy not only for limiting the progression of heart failure but also for controlling the development of arrhythmias.

In animal models of PH, the contribution of oxidative stress to cardiac dysfunction and myocyte apoptosis is detected [88,89,90]. Through the inhibition of MLCP and of co-repressor cardiac ankyrin-repeat protein (CARP), ROCK stimulates the transcriptional activity of the ventricular isoform of myosin light chain (MLC-2v), inducing cell apoptosis and increasing NOX-2 content and reactive oxygen species generation in H9c2 cardiac myoblasts and rat hearts after hypoxic injury [81,89]. Interestingly, both the ROCK inhibition of eNOS and cardiac oxidative stress result in less cyclic GMP generation, hindering the PKG inhibition of RhoA and further activating ROCK in a positive feedback loop [90,91]. The exacerbation of oxidative stress may lead to irreversible cell damage and trigger cell death mechanisms in cardiomyocytes [74,88,92,93,94,95]. Although both ROCK isoforms contribute to cardiac apoptosis, it is a strong relation with ROCK1 activity, as selective downregulation prevents the transition from compensatory hypertrophy to heart failure [24,38].

### 3.4. Fibroblasts

Pulmonary adventitial fibroblasts can be activated in response to common insults involved in PH, including mechanical stress, hypoxia or inflammatory cytokines. These stimuli provoke phenotypic changes in fibroblasts, including not only an increase in their proliferation and migratory potential, but also inducing the production of cytokines and adhesion molecules and enhancing their capacity of extracellular matrix turnover [43,44]. This activated phenotype promotes the recruitment of leukocytes to the vascular wall, modulates immune responses and may underlie the genesis of pulmonary hypertension secondary to lung or connective tissue diseases [43]. Moreover, activated fibroblasts are also responsible for RV changes in PH, especially the fibrotic remodeling of myocardial matrix after mechanical overload [23].

In cardiac and pulmonary tissues, the pro-fibrotic role of ROCK involves the transdifferentiation of fibroblasts into myofibroblasts [23,39,96,97]. Enhanced ROCK activity on fibroblasts in cardiovascular diseases is responsible for the de novo expression of a pro-fibrotic gene program through the TGF-β, MRTF/serum response factor (SRF) and YAP/TAZ pathways [23]. In contrast, ROCK inhibitors limit the progression of cardiac remodeling and fibrosis after angiotensin-II or *N*^ω^-nitro-L-arginine methyl ester (L-NAME) treatment [23]. Although the implications of both ROCK isoforms in tissue fibrosis, transgenic mice studies revealed that *ROCK1* knockout or knockdown led to a marked attenuation of cardiac extracellular matrix remodeling in response to angiotensin-II, pressure overload or myocardial infarction, while *ROCK1* overexpression caused spontaneous cardiac fibrosis [23,38,39,76]. The ROCK2 isoform in cardiac fibroblasts increases the expression of pro-fibrotic genes in the heart [98].

In addition to their role in fibroblast phenotype modulation, both ROCK isoforms also regulate its migration, proliferation and apoptosis resistance. The use of ROCK inhibitors demonstrated the crucial participation of mechano-transduction in lung and cardiac fibroblasts, promoting tissue invasion by modulating focal adhesions composition and increasing the secretion of matrix metalloproteinases [99,100,101]. Moreover, ROCK activity is also required for epithelial-to-mesenchymal transition in lung epithelial cells and cardiac epicardial-derived stem cells, which may contribute to an increased fibroblast population and tissue fibrosis [102,103].

Lung fibroblasts isolated from patients with idiopathic pulmonary fibrosis also display increased resistance to apoptosis, a phenotype which reflects the higher expression of B cell lymphoma (Bcl)-2 in these cells by activation of the ROCK/MRTF/SRF pathway [104]. The exposure of fibroblasts to hypoxia leads to *ROCK1* gene upregulation, which further contributes to increased migration, proliferation and phenotypic plasticity in those cells by crosstalk between ROCK1 and HIF-1α [105,106]. Recently, a growth factor-independent pathway for fibroblast activation and differentiation was described, which relies on the activation of the signal transducer and activator of transcription (STAT)-3 by ROCK and may contribute to the exacerbation of tissue fibrosis [107].

### 3.5. Leukocytes

Vascular inflammation is acknowledged as a major player in PH pathophysiology, as increased incidence of PH is found in patients with infectious (schistosomiasis, HIV) or autoimmune diseases (systemic lupus erythematosus, scleroderma). Remodeled vessels seen in humans and rodents with PH display extensive perivascular leukocyte infiltrate, mainly constituted by monocytes/macrophages, dendritic cells, lymphocytes and mast cells [43,44,108,109]. Thus, the activation of immune cells is also implicated in RV remodeling and dysfunction in PH [52,110].

The inhibition of ROCK isoforms has demonstrated potent anti-inflammatory effects on preclinical models of cardiovascular and autoimmune diseases, by modulating the migration and activation of leukocytes [27,111,112,113,114]. The increased ROCK content found in circulating leukocytes from PH patients [115] may be a predictor of cardiovascular event risk, as observed in cardiac diseases [116,117,118,119]. Moreover, a positive correlation was demonstrated between ROCK activity in circulating leukocytes and cardiac/vascular tissues in rats [120], indicating its direct influence on immune cell activation in cardiovascular diseases.

The activation of ROCK may contribute to increased leukocyte recruitment to vascular lesions by modulating the contractility of the actomyosin cytoskeleton and promoting cell adhesion and extracellular matrix invasion [27,121,122]. The increase in those effects is observed in monocytes of stiffer matrix and may contribute to increased migration in sites of active collagen synthesis or limited disruption of the endothelial barrier [61,122,123]. The selective knockdown of ROCK1 increases the migration of macrophages in vivo, which indicates the more prominent role for ROCK2 in mediating myeloid cell recruitment to extravascular sites [113,124]. Tissue invasion by pro-inflammatory lymphocytes also contributes to vascular remodeling in PH and the activation of intracellular ROCK increases the migratory velocity of these cells in collagen gels in response to chemotactic stimuli [125,126].

#### 3.5.1. Macrophages

The most abundant inflammatory cells found in the pulmonary vasculature of animals and humans with PH are macrophages, which appear in an early stage of vessel remodeling and their accumulation persists during disease progression [43,108]. The participation of macrophages in PH development was suggested by the attenuated pulmonary arterial pressure and RV systolic pressure in rodent models of PH after lung macrophage depletion [43,127]. Moreover, the phenotypic plasticity of macrophages seems to be implicated in different contributions to PH pathophysiology, with the early predominance of a pro-inflammatory, pro-apoptotic M1 phenotype and the progressive change towards a pro-proliferative, pro-fibrotic M2 phenotype during disease progression. These changes were recently described in the lungs of a rat model [128] and are supposed to modulate cardiac injury and fibrosis in the RV [110].

The inhibition of ROCK impairs the adhesion of peripheral blood monocytes to endothelial cells [124], and reduces their migration, proliferation and differentiation [113,129,130], reflecting the relevance of the enzyme to mononuclear cell infiltration in PH lungs [131,132]. The activation of ROCK also mediates macrophage biomechanical functions with the maintenance of cell shape and the stimulation of amoeboid migration [113,133,134,135]. Additionally, it also blocks the macrophage phagocytosis of apoptotic cells [136,137,138,139], which serves as a signal for polarization towards the M2 phenotype, for the resolution of inflammation. However, polarization to M2 macrophages is induced by ROCK when cells make contact with matrices of high anisotropy and/or medium stiffness [140,141], such as newly synthesized collagen fibers deposited by activated fibroblasts, which may indicate a positive feedback of tissue fibrosis signaling. These effects show some dependency on the ROCK isoform, since ROCK1 produces an inflammatory M1 phenotype while ROCK2 increases the expression of M2 markers in mice models of inflammation [23,113,142].

#### 3.5.2. Mast Cells

Mast cells are also profoundly implicated in human and rodent PH [109,143], by releasing multiple mediators involved in endothelial permeability, angiogenesis, smooth muscle proliferation and immune cell stimulation [43,144,145,146,147,148]. Mast cell proteases (tryptase and chymase) contribute to PH progression and RV remodeling through the activation of vascular and cardiac stromal cells or by increasing local production of angiotensin-II, endothelin-1, interleukins and growth factors, further enhancing hypertrophic and fibrotic stimuli [44,110,149,150,151]. Although studies on the influence of ROCK on mast cell biology are limited, it was recently reported that ROCK1 activation not only mediates their adhesion and migration but also promotes proliferation, maturation and degranulation [152,153].

#### 3.5.3. Neutrophils

Despite the implication in early phases of tissue inflammation, the contribution of neutrophils to PH has only recently become a subject of interest. Neutrophils are found in perivascular infiltrates in rodent models and human PH with increased ROCK activity although their lower level in PH lungs [43,109]. However, the blood neutrophil/lymphocyte ratio shows a positive correlation to vascular resistance and PH functional class, which could predict survival [43,154,155]. Although it mediates cell polarization and increases the migratory velocity in these cells [121,156,157], a negative impact of ROCK activation is reported on neutrophil functions, such as phagocytosis, the production of superoxide and adhesion to activated endothelial cells [158,159,160]. ROCK would limit the extravasation of neutrophils to the perivascular space and contribute to vascular inflammation from the luminal side. In fact, myeloperoxidase and neutrophil elastase are implicated in hypoxic vasoconstriction and vascular remodeling through the modulation of smooth muscle and endothelial cells in rodent models of PH and are currently subjects of clinical investigation [43,155,161].

#### 3.5.4. Dendritic Cells

The appearance of perivascular tertiary lymphoid follicles is a histopathological feature found along remodeled vessels in lungs of idiopathic PH patients [11]. These structures contain different cell types engaged in adaptive immune responses around the vasculature and may provide a link between autoimmunity and tissue remodeling. The activation of the adaptive immune system relies on antigen presentation by phagocytic cells (dendritic cells and macrophages), which migrate from the site of inflammation to lymphoid tissues. The accumulation of dendritic cells on the vicinity of vessels in PH is reported [43,109,162], and their contribution to disease progression may depend on the ROCK modulation of dendritic cell activation, migration and phagocytosis [27,121,162,163].

#### 3.5.5. T and B Lymphocytes

The increased number of activated T cells found in lung vessels from patients and animals with PH demonstrates the importance of immune dysregulation to the pathology of the disease [43,109]. Vascular remodeling is stimulated in different conditions by CD4 T helper cells with T_h_1, T_h_17 or T_h_2 phenotypes, suggesting an imbalance of stimulatory and inhibitory mechanisms of immune cell activation in disease progression [11,164]. In contrast, regulatory T cells (T_reg_) are implicated in suppressing immune responses on the vessel wall indicating anti-inflammatory role in PH [43,109,165]. In addition to the mediation of lymphocyte chemotaxis, ROCK activity also regulates T cell activation, proliferation and cytokine production [27,121,166,167,168]. Isoform-selective responses are suggested, because ROCK1 and ROCK2 modulate the polarization of CD4 T cells to T_h_2 and T_h_1/T_h_17 phenotypes, respectively [27,113,167,169]. ROCK2 inhibition reduces the phosphorylation of STAT-1 and STAT-3 and increases phosphorylated STAT-5, thus favoring T_reg_ polarization instead of T_h_1/T_h_17 and locally controlling immune cell activation [126,170].

Activated B cells in lymphoid tissues are responsible for antibody production and antigen presentation to T cells and are associated with autoimmune responses in PH [43]. Currently, the role of ROCK on B cell functions remains poorly addressed. Despite the essential role of RhoA for the survival and development of B cells, this effect does not seem to rely solely on ROCK activity [166]. In activated B cells, ROCK mediates the antiapoptotic effect of type I interferon [171], while ROCK inhibition reduces proliferation and promotes apoptosis in transformed B cells [172,173]. Recently, a role for ROCK2 in controlling B cell proper location inside germinal centers in mice and humans was suggested, presumably by activating PTEN and thus, reducing Akt inhibition on forkhead box protein O1 (FOXO1) [174,175]. The activation of ROCK1 also seems to be required for antigen internalization through B cell receptors [166], which would prove useful to PH caused by scleroderma or systemic lupus erythematosus.

### 3.6. Platelets and Red Blood Cells

Although platelets and red blood cells display important roles in pulmonary hypertension associated with hypoxic, thromboembolic or hematologic diseases [176,177,178], their contribution to PH pathophysiology remains poorly understood. Both cell types may interfere with disease progression and RV dysfunction because they impair pulmonary hemodynamics and promote cardiovascular remodeling [179].

#### 3.6.1. Platelets

The activation of ROCK mediates shape change and aggregation, clot contraction and ATP secretion in response to agonist stimulation of human and rodent platelets [180,181,182]. These effects are mainly dependent on ROCK2, as recently demonstrated in platelet-selective *ROCK2*-knockout mice [183]. Shape change is markedly enhanced by a 2.7-fold higher ROCK activity found in platelets in women than men [184], which may be of relevance given the greater prevalence of PH among women. Although this issue remains controversial [185], the sex-specific differences in ROCK activity in platelets from PH patients should be considered.

Platelet cytoplasmic granules contain important mediators implicated in proliferative and inflammatory responses on the vasculature. Serotonin is stored in dense granules, which is a key mediator of VSMC proliferation and is increased in platelets of PH patients with increased ROCK activity [185]. ROCK mediates the release of PDGF and P-selectin from the α-granules of activated platelets, which induces VSMC hyperplasia and monocyte transendothelial migration, respectively [180,181,186]. Hence, the inhibition of ROCK by Y-27632 diminishes superoxide production by NADPH oxidase in human platelets activated by agonists of PAR or the thromboxane receptor [180] and may interfere with endothelial cell function by reducing oxidative stress and increasing NO bioavailability.

#### 3.6.2. Red Blood Cells

Red blood cell distribution has been identified as a biomarker for predicting mortality, response to treatment and incidence of heart failure in patients with PH [187,188]. Increased pulmonary vascular resistance is associated with red blood cell stiffness in mice and ROCK regulates the level of abnormal erythrocyte [189,190]. Red blood cells from PH patients were found to have increased ROCK content, which was also correlated to their lower endothelial NOS activity, because treatment with fasudil recovered the NO production [191,192]. ROCK may reduce ATP release from human erythrocytes under hypoxic conditions [190], thus impairing the purinergic stimulation of endothelium-dependent vasodilator responses and favoring hypoxic pulmonary vasoconstriction.

**Figure 1:**
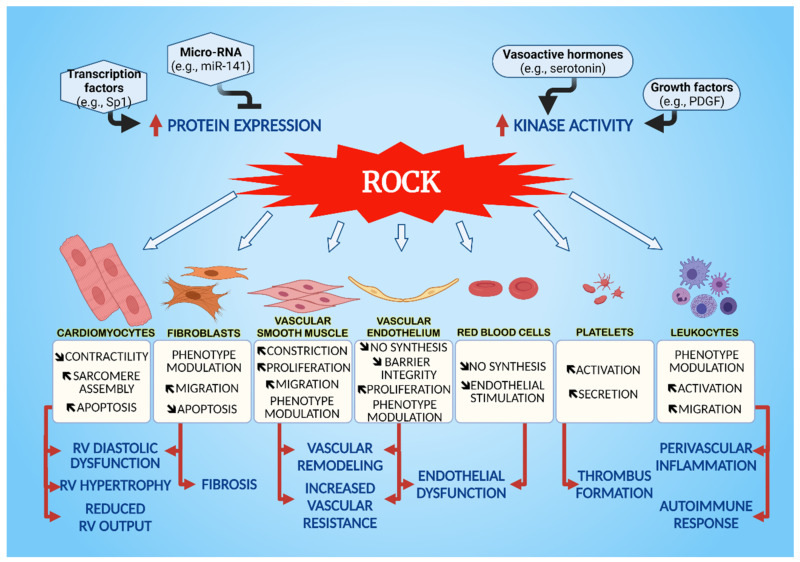
Influence of ROCK overexpression and activation on the main cellular effects contributing to PH pathophysiology.

The pathophysiology of PH involves distinct contributions from endothelial and smooth muscle cells in order to maintain the sustained vasoconstriction and proliferation. Hemodynamic alterations profoundly affect cardiomyocyte structure and metabolism, leading to hypertrophic and apoptotic responses involved in RV failure. Fibroblasts are associated with cardiac and perivascular fibrosis and immune cell recruitment and activation further stimulates tissue remodeling. ROCK integrates important signaling events mediating changes in cell structure and function implicated in disease progression and, therefore, represents an important drug target for treatment of PH (Figure 1).

## 4. ROCK in Preclinical Models of PH

Strong evidence from cell studies indicates that ROCK is activated [19] and contributes to PH pathogenesis and its modulation could interfere with the alterations induced in different PH experimental models, using monocrotaline (MCT) [49,193,194,195,196,197,198,199,200,201,202,203] or hypoxia [35,126,162,198,202,204,205,206].

Fasudil is the first described and only clinically available ROCK inhibitor [207]. The long-term inhibition of ROCK by fasudil prevents and promotes the improvement of MCT-induced PAH through the inhibition of VSMC proliferation with increased apoptosis and reduced macrophage infiltration, resulting in improved endothelium-dependent relaxation and VSMC contraction [200]. When orally administered, fasudil reduces RV systolic pressure (RVSP) and improves pulmonary vascular remodeling in PH induced by MCT or chronic hypoxia, attenuating oxidative stress by increasing the concentration of superoxide dismutase (SOD) and reducing levels of H_2_O_2_, malonyldialdehyde (MDA) and hydroxyl radical in addition to inhibiting the pulmonary expression of thioredoxin-1 (Trx1) and hypoxia-inducible factor-1α (HIF-1α) [202]. The effects of fasudil in MCT-induced PH are partially mediated by the reduction in nerve growth factor (NGF) signaling [203] that promotes the proliferation and migration of vascular cells, as well as increases the secretion of pro-inflammatory cytokines in the pulmonary arteries. The involvement of NGF in PH induced by chronic hypoxia or MCT was demonstrated with the reversal of vascular alterations after treatment with an anti-NGF antibody [208].

In PH, there is an increase in lysophosphatidic acid (LPA), a potent activator of RhoA/ROCK signaling [205], which contributes to the remodeling of the pulmonary vasculature [209]. The exogenous administration of LPA exacerbates mPAP, pulmonary and cardiac vascular remodeling in hypoxia-induced PH in rats with T_h_17/T_reg_ cell imbalance, which is reverted with fasudil [126].

In addition to fasudil, several substances described as ROCK inhibitors have been investigated (Table 1) [49,195,196,197,198,199,201]. Recently, fasudil dichloroacetate (FDCA) was synthesized and demonstrated a preserved inhibitory profile of ROCK2 [201]. FDCA reduces PH-induced TNF-α and IL-6 release in pulmonary artery endothelial cell culture (PAEC) and pulmonary artery smooth muscle (PASMC). Additionally, FDCA showed better results than fasudil in reducing the mean pulmonary arterial pressure, RVSP and showed similar results concerning the improvement of pulmonary vascular remodeling, RV hypertrophy and collagen deposition index when administered orally for 14 days [201].

Other ROCK inhibitors, azaindol-1 [198], SB-772077-B [210] and KD025 [211], decrease pulmonary and systemic arterial pressures in rodents with MCT-induced PH [199] and RV hypertrophy, pulmonary resistance, muscularization and pulmonary vasculature thickness. When compared to macitentan [196], Y-27632, an inhibitor of both ROCK1 and ROCK2 [207], showed better effects in reducing RVSP and RV remodeling. However, the use of Y-2763 is associated to a significant reduction in systemic blood pressure [196]. Fasudil (30 mg/kg) and Y-27632 (15 mg/kg) improve RV remodeling with reduced RVSP, RV hypertrophy and thickness of the wall of the pulmonary arterioles, without modifying the expression of ROCK1 and ROCK2 that are elevated in animal model PH. In contrast, the pulmonary arteries isolated from animals treated with fasudil or Y-27632 showed a reduced contractile response of pulmonary arteries, suggesting that the inhibition of the RhoA/ROCK signaling pathway regulates storage-operated Ca^2+^ channels (SOCCs) and receptor-operated Ca^2+^ channels (ROCCs) modulating pulmonary artery contractility [35]. Fasudil or Y-27632 attenuated the increased intracellular Ca^2+^ in PASMC induced by PH, providing support for the statement that the RhoA/ROCK pathway plays a role in the pulmonary Ca^2+^-dependent vasoconstriction [35].

**Table 1:** ROCK inhibitors other than fasudil and pulmonary hypertension.

Compound	Model	Effect	Reference
FDCA	Hypoxia in PAEC PASMC	↓ TNF-α	[201]
	PDGF-BB in PAEC/PASMC	↓ TNF-α ↓ IL-6	
	MCT-induced PH	↓ mean PAP ↓ RVSP ↓ RV hypertrophy ↓Pulmonary vascular remodeling ↓ RV hypertrophy ↓ Collagen RV	
SB772077-B	MCT-induced PH	↓ PAP ↓ systemic arterial pressures	[199]
Azaindol-1	PH induced by MCT or chronic hypoxia	↓ RVSP ↓RV hypertrophy ↓ Pulmonary resistance ↓ muscularization ↓ pulmonary vasculature thickness ↓ expression of p-MYPT1, ↓ PCNA-positive vascular cells in the lungs	[198]
KD025	PAH induced by MCT	↓ RVSP	[211]
Y-27632	PAH induced by MCT	↓ RVSP ↓ RV remodeling ↑ Cardiac output	[196]
	PAH induced by chronic hypoxia	↓ RVSP ↓ RV hypertrophy ↓ PA wall thickness ↓ contraction in isolated PA	[35]
		↓ RVSP	[162]
	Hypoxia in PASMC	↓ [Ca^2+^]_i_ Suppression of SOCE and ROCE ↓ Expression of TRPC1, TRPC2, HIF-1α	[35]
Compound 3 *	MCT-induced PH	Improves hemodynamics ↓ Vascular remodeling	[197]
Aloperine	MCT-induced PH	Improvement in hemodynamics ↓ Cardiac hypertrophy ↓ Pulmonary vascular remodeling ↓ Expression protein RhoA, ROCK1 and ROCK2 ↑ Expression protein p27^kip1^ and Bax ↓ Activation of MYPT1	[195]
18β-GA	MCT-induced PH	Improvement in hemodynamics ↓ Cardiac hypertrophy ↓ Pulmonary vascular remodeling	[49]
	PDGF-BB in hPASMC	↓ Expression protein RhoA, ROCK1 and ROCK2 ↑ Expression protein p27^kip1^ and Bax ↓ Activation of MYPT1	[49]

18β-GA, 18β-glycyrrhetinic acid, FDCA, fasudil dichloroacetate, HIF-1α, hypoxia-inducible factor 1α, hPASMC, human pulmonary artery smooth muscle cells, IL-6, interleukin-6, MCT, monocrotaline, PAEC, pulmonary arterial endothelial cell culture, PAP, pulmonary arterial pressure, PASMC, pulmonary artery smooth muscle, PDGF-BB, growth factor derived from BB platelet, PH, pulmonary hypertension, ROCE, receptor-operated Ca^2+^ entry, RV, right ventricular, RVSP, right ventricular systolic pressure, SOCE, store-operated Ca^2+^ entry, TNF-α, tumor necrosis factor, TRPC, transient receptor potential canonical channel. * Compound 3: *trans*-6-((4-aminocyclohexyl)amino)-5-fluoro-2-methoxynicotinamide.

The female PL/J mouse strain shows extremely high RVSP after exposure to chronic hypoxia with the association of ROCK2 activation. Since fasudil or Y-27632 reduces RVSP by approximately 30%, it is safe to assume that ROCK inhibition should be involved in the hemodynamic alterations [162]. Compound 3 (*trans*-6-((4-aminocyclohexyl)amino)-5-fluoro-2-methoxynicotinamide) was ten and sixty times more potent than Y-27632 and fasudil, respectively, as an inhibitor of ROCK1 and ROCK2 [197]. In rodents with MCT-induced PH, this substance improves hemodynamics and vascular remodeling without affecting heart rate and systolic blood pressure [197]. However, Compound 3 does not provide protection for RV hypertrophy, which is different from that observed for fasudil [212].

Aloperine (a component of *Sophora flavescens* Ait.) and 18β-glycyrrhetinic acid (18β-GA, a bioactive component of *Glycyrrhiza glabra* L.) produce anti-inflammatory and antioxidant effects. For that reason, they were tested in MCT-induced PH and both aloperine [195] and 18β-GA [49] promoted protective with improvement in hemodynamics, cardiac hypertrophy and the attenuation of pulmonary vascular remodeling, possibly due to RhoA/ROCK regulation because of the reduction in protein and mRNA expression for RhoA, ROCK1 and ROCK2 [195].

The polymerization of actin also contributes to the increase in pulmonary vascular reactivity in PH. Cytochalasin (CytB, an actin polymerization inhibitor), fasudil, tiron (a reactive oxygen species scavenger) and SMIFH2 (an inhibitor of formin homology domain 2) blocked the increase in endothelin-1-induced actin polymerization in pulmonary arteries isolated from rats exposed to chronic hypoxia. Thus, interference in the RhoA/ROCK pathway can reduce the intense vasoconstriction of pulmonary vessels [206].

The gene silencing of *ROCK2* by small interfering RNA (siRNA) in primary pulmonary artery smooth muscle cell culture from patients with PH (PH-PASMC) induces a reduction in cell migration and proliferation [204] indicating that ROCK2 contributes to the progress of PH. The activation of the RhoA/ROCK pathway also occurs in cultured human pulmonary arterial smooth muscle cells (hPASMC) exposed to hypoxia [194].

Therefore, by using different cellular and animal models, the above studies indicate that ROCK activation is closely involved in the development of cardiac and vascular alterations in PH. In addition, ROCK inhibitors, especially those with greater ROCK2 selectivity, provided improvement in vascular remodeling and cardiac dysfunction in preclinical models, which highlighted the role of ROCK as drug target for PH and prompted the subsequent clinical evaluation of ROCK inhibition for its treatment.

## 5. Clinical Trials of ROCK Inhibitors in PH

Since basic science investigations suggested that ROCK inhibitors produce multiple beneficial effects in the cardiovascular system, ROCK is an alternative target to ameliorate cardiac and vascular dysfunction in cardiopulmonary diseases. Besides a direct vasodilatory effect, the inhibition of ROCK would also prevent the tissue remodeling and the progression of PH. However, despite many clinical trials providing the basis of the promising use of ROCK inhibitors to treat cardiac diseases, currently no ROCK inhibitor has been approved for PH treatment.

Fasudil has been used for the prevention and treatment of cerebral vasospasm after subarachnoid hemorrhage surgery since 1994. The unique clinically approved ROCK inhibitor, fasudil, has been extensively used in clinical trials in order to evaluate the influence of ROCK in cardiovascular diseases, such as vasospastic angina, coronary artery disease and PH [213]. Regarding PH, several short-term clinical studies have been conducted since 2005 and have demonstrated an important reduction in pulmonary vascular resistance and improvement in the RV cardiac index by the intravenous infusion of fasudil (30 or 60 mg over 30 min) in patients with PH from both sexes, without a significant reduction in systemic blood pressure or heart rate [213]. In a recent randomized, controlled, crossover study, the comparison of fasudil intravenous infusion and inhaled iloprost revealed a significant improvement in RV cardiac output and oxygen saturation by fasudil, although no differences in the reduction in pulmonary vascular resistance were observed between both agents [214]. Importantly, most acute studies demonstrated the selectivity of fasudil for vasodilation in the pulmonary vascular bed, because systemic arterial pressure changes were not detected, even at 60 mg [213,215]. Minor side effects reported included facial flushing, headache, xerostomia and transient abdominal pain during fasudil infusion.

When used as add-on to standard PH therapy, intravenous fasudil (30 mg) for a median duration of 8 days provided a significant reduction in both in-hospital mortality and 30-day re-hospitalization when compared to standard therapy [213]. Considering the PH secondary to left ventricle heart failure, introduction of intravenous fasudil together with the standard therapy, significantly improved exercise tolerance and serum levels of NT-pro brain natriuretic peptide although a reduction pulmonary artery systolic pressure and left ventricle diastolic function was observed only in patients with reactive PH [216]. One clinical study observed the benefit of oral fasudil therapy in a 3-month double-blinded, randomized, placebo-controlled, multicenter trial. In this study, fasudil improved cardiac index after 12 weeks, although no significant changes in pulmonary vascular resistance was observed, explained by the reduced number of patients [216].

Despite the encouraging results in acute treatment, no clinical trial has been con-ducted to study the long-term effects of fasudil therapy in PH. Fasudil displays low inhibi-tory potency [217], which implicates in the clinical use of high dose that may increase ad-verse effects consequent to non-selective inhibition of other protein kinases. Oral admin-istration of fasudil in PH patients [218], demonstrated its low bioavailability, since it is rapidly metabolized by aldehyde oxidase (AOX) [219]. Differences in AOX expression and activity between species lead to distinct pharmacokinetic profile and are currently consid-ered responsible for the failure of Phase 1 studies of protein kinase inhibitors [219,220,221]. The impact of AOX gene polymorphisms on enzyme activity in humans [221] may also produce erratic results in treated groups. These features of oral administration of fasudil prompted further search for new ROCK inhibitors [222] and improvement of pharmacoki-netic profile is a major need.

Therefore, although most trials highlight the relevance of ROCK inhibition for the treatment of PH, additional clinical studies should be conducted in order to ascertain the long-term effects of this approach. The oral therapy of fasudil is strongly suitable and should be evaluated in order to determine the benefits for patients with heart diseases.

## 6. Conclusions

PH remains a disease of high morbidity and mortality, despite the currently available drugs, which ameliorate pulmonary vasoconstriction and RV afterload. Preclinical and clinical studies highlighted the importance of addressing the proliferative and inflammatory components of PH, changing the current strategy for the treatment of this disease. ROCKs are important protein kinases involved in multiple cell processes, such as the regulation of cell cycle, apoptosis, motility, adhesion, the contraction and modulation of gene expression and cell phenotype in response to agonist stimulation and changes in extracellular matrix arrangement or mechanics. These events intimately related to vascular and cardiac remodeling can account for important alterations seen in many cardiovascular diseases such as PH. Preclinical studies using MCT- or hypoxia-induced PH in rodent models evidenced the beneficial role of ROCK inhibition in reducing the proliferative vascular lesions in lungs, while reducing pulmonary vascular resistance and preventing RV dysfunction, hypertrophy and fibrosis. Additionally, research using the clinically approved drug fasudil provided further evidence for the selective reduction in vascular resistance in short-term studies in patients with PH. Finally, there is compelling evidence that ROCK is a promising drug target for reducing vascular remodeling and cardiac dysfunction progression, which may increase survival in PH patients.
